# The Rendu-Osler-Weber Disease Revealed by a Refractory Hypoxemia and Severe Cerebral Fat Embolism

**DOI:** 10.1155/2013/434965

**Published:** 2013-08-01

**Authors:** Leonel Barreto, Jean-Bernard Amiel, Anthony Dugard, Nicolas Pichon, Marc Clavel, Bruno François, Philippe Vignon

**Affiliations:** ^1^Medical-Surgical ICU, Dupuytren Teaching Hospital, 87000 Limoges, France; ^2^CIC-P 0801, Dupuytren Teaching Hospital, 87000 Limoges, France; ^3^University of Limoges, 87000 Limoges, France; ^4^Service de Réanimation Polyvalente, CHU Dupuytren, 2 Avenue Martin Luther King, 87042 Limoges Cedex, France

## Abstract

The Rendu-Osler-Weber disease is a genetic disease which may lead to severe hemorrhage and less frequently to severe organ dysfunction. We report the case of a 22-year-old patient with no personal medical history who was involved in a motorcycle accident and exhibited severe complications related to large arteriovenous pulmonary shunts during his ICU stay. The patient developed an unexplained severe hypoxemia which was attributed to several arteriovenous shunts of the pulmonary vasculature by a contrast study during a transesophageal echocardiographic examination. The course was subsequently complicated by a prolonged coma associated with hemiplegia which was attributed to a massive paradoxical fat embolism in the setting of an untreated femoral fracture. In addition to hemorrhagic complications which may lead to intractable shock, arteriovenous malformations associated with the Rendu-Osler-Weber disease may involve the pulmonary vasculature and result in unexpected complications, such as hypoxemia or severe cerebral fat embolism in high-risk patients.

## 1. Introduction

Although epistaxis is usually the first symptom of the Rendu-Osler-Weber disease, the severity of vascular lesions and related organ dysfunctions increases with age [1, 2]. We report the case of a young blunt trauma patient whose disease was revealed by life-threatening complications related to large pulmonary arteriovenous shunts.

## 2. Case Report

**Figure 1:**
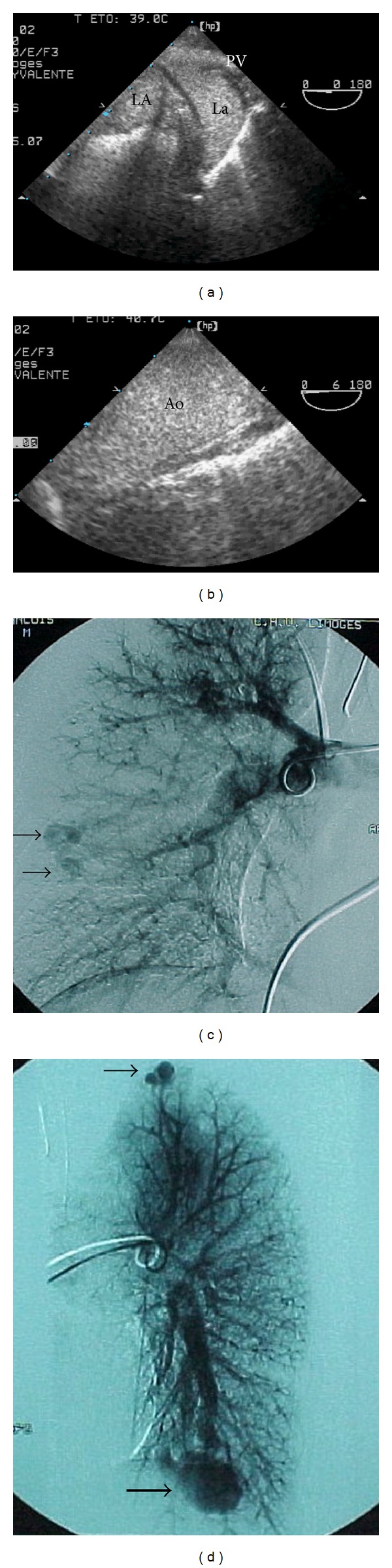
Pulmonary arteriovenous fistulae associated with the Rendu-Osler-Weber disease. The contrast study performed during a transesophageal echocardiographic examination depicted a massive opacification of the left atrium a few beats after the opacification of the right atrium through the left and right pulmonary veins (a), but no patent foramen ovale, and a subsequent massive opacification of the aortic arch (b). Pulmonary angiography confirmed the presence of multiple arteriovenous fistulae in the two lungs ((c) and (d), arrows), the largest being located in the left inferior lobe (thick arrow). These lesions were subsequently excluded from the pulmonary circulation by serial percutaneous transcatheter embolizations. LA: left atrium; La: left atrial appendage; PV: pulmonary vein; Ao: aortic arch.

A 22-year-old motorcyclist without medical history was involved in a violent head-on collision. The patient was initially conscious; he had no motor deficit and no hemodynamic or respiratory compromise. Contrast-enhanced body CT scan ruled out a head trauma but disclosed multiple facial fractures, a mandibular fracture, a rounded opacity in the left pulmonary base consistent with an arteriovenous shunt, a hepatic contusion, a fractured left iliac crest, and a closed fracture of the right femoral diaphysis. The patient was promptly referred to the operating suite where his facial wounds were sutured and a maxillomandibular fixation was placed. The femoral osteosynthesis was postponed due to unstable hemodynamics, and the right lower limb was immobilized with a traction. Hemodynamics were stabilized through the transfusion of red cells and plasma units. The patient was admitted to the ICU with normal blood pressure (120/75 mmHg), body temperature (36.7°C), and blood oxygenation (SpO_2_: 99%). An abrupt hypotension occurred (60/35 mmHg) in conjunction with sinus tachycardia (130 bpm), hyperthermia (40°C), and marked oxygen desaturation (SpO_2_: 81%). Patient's level of consciousness rapidly deteriorated and a petechial rash was noted in the deltopectoral triangle and left eye conjunctiva. There was no evidence of sepsis and blood cultures remained sterile. Biological tests revealed the presence of a coagulopathy (prothrombin time: 46%; platelet count: 67,000/mm^3^; fibrinogen: 1.5 g/dL) but haemoglobin level was preserved (10.8 g/dL). A vasopressor support was initiated and hypoxemia persisted despite a 100% FiO_2_ and a PEEP challenge (PaO_2_/FIO_2_: 110). Abdominal ultrasound and bedside chest X-ray were unremarkable. Transesophageal echocardiography (TEE) ruled out a traumatic disruption of the aorta and depicted a normal heart. A contrast study excluded a patent foramen ovale but disclosed a massive anatomical shunt of the pulmonary vasculature (Figure 1). Pulmonary angiography depicted a large left basal arteriovenous fistula and three other vascular shunts of smaller size (Figure 1). Serial percutaneous transcatheter embolizations were successfully performed. Rapid improvement of blood oxygenation was observed (PaO_2_/FIO_2_: 360). The patient remained comatose with a left hemiplegia. Head CT scan disclosed multiple areas of brain infarction in the right posterior temporal area. Brain magnetic resonance imaging confirmed the presence of ischemic areas located in the right boundaries between the frontal and the parietal areas, associated with a limited infarction confined to the left parietal cortex. The femoral osteosynthesis was performed and as the patient progressively recovered normal consciousness, he was weaned from a ventilator on Day 15. The patient was then discharged from the ICU on Day 23. Further investigations revealed a familial history of the Rendu-Osler-Weber disease, with recurrent severe epistaxis in the patient's father.

## 3. Discussion

In the present case, the Rendu-Osler-Weber disease was fortuitously identified in a trauma patient secondary to severe complications related to large pulmonary anatomic shunts. These complications led to a sustained hypoxemia under ventilator and have presumably facilitated cerebral fat embolism with secondary neurological compromise. Noticeably, the two complications had an abrupt onset and developed concomitantly once the patient had been stabilized.

Unexplained (absence of radiographic infiltrates), sustained (negative recruitment maneuvers) hypoxemia under ventilator should raise the diagnosis of an anatomical shunt, mostly intracardiac (i.e., patent foramen ovale) or rarely intrapulmonary [3]. TEE contrast study is the reference imaging modality to depict central anatomical shunts [4]. In our patient, the chest CT scan had initially disclosed potential pulmonary arteriovenous malformations. Serial embolization procedures resulted in a dramatic improvement of blood oxygenation so that the patient could be successfully weaned from the ventilator.

Although fat embolism is challenging to recognize in ventilated trauma patients, our patient met all major clinical criteria [5]: petechiae in the anterior thorax, thrombopenia, high body temperature, and coagulation disorder in the absence of hemorrhagic shock. Massive cerebral fat embolism facilitated by an untreated femoral fracture in conjunction with large pulmonary arteriovenous malformations presumably accounted for the neurological compromise of our patient who had no head trauma. Cerebral CT scan and MRI depicted multiple ischemic areas which failed to match cerebral artery territories.

Only 5 to 15% of patients with the Rendu-Osler-Weber disease exhibit anatomical shunt of the pulmonary vasculature [2]. This genetic disease is rarely diagnosed in the ICU settings. In addition to hemorrhagic complications that may lead to intractable shock, multiple arteriovenous malformations may involve the pulmonary vasculature and result in unexpected complications such as unexplained hypoxemia and fat cerebral embolism.
